# Signal envelope and speech intelligibility differentially impact auditory motion perception

**DOI:** 10.1038/s41598-021-94662-y

**Published:** 2021-07-23

**Authors:** Michaela Warnecke, Ruth Y. Litovsky

**Affiliations:** University of Wisconsin-Madison, Waisman Center, 1500 Highland Ave, Madison, WI 53705 USA

**Keywords:** Psychology, Human behaviour

## Abstract

Our acoustic environment contains a plethora of complex sounds that are often in motion. To gauge approaching danger and communicate effectively, listeners need to localize and identify sounds, which includes determining sound motion. This study addresses which acoustic cues impact listeners’ ability to determine sound motion. Signal envelope (ENV) cues are implicated in both sound motion tracking and stimulus intelligibility, suggesting that these processes could be competing for sound processing resources. We created auditory chimaera from speech and noise stimuli and varied the number of frequency bands, effectively manipulating speech intelligibility. Normal-hearing adults were presented with stationary or moving chimaeras and reported perceived sound motion and content. Results show that sensitivity to sound motion is not affected by speech intelligibility, but shows a clear difference for original noise and speech stimuli. Further, acoustic chimaera with speech-like ENVs which had intelligible content induced a strong bias in listeners to report sounds as stationary. Increasing stimulus intelligibility systematically increased that bias and removing intelligible content reduced it, suggesting that sound content may be prioritized over sound motion. These findings suggest that sound motion processing in the auditory system can be biased by acoustic parameters related to speech intelligibility.

## Introduction

The dynamic and noisy nature of our everyday environment consists of a multitude of sound sources that vary perpetually in acoustical features. The auditory scene is further complicated by the fact that sound sources are not stationary; in fact, sounds typically move, either because the listener moves, or the sound source does. Listeners analyze the auditory environment to successfully navigate it, in part by localizing sounds, identifying their sources and content, and by segregating meaningful sounds from background noise^1^. Humans commonly allocate attentional resources to a location in space following the appearance of a sound, underscoring the fact that spatial attention plays a crucial role in both extracting auditory information from meaningful target sounds^2,3^ and detecting sound motion in space^4,5^.

Meaningful target sounds often contain speech, which consists of varying temporal and spectral information, and can be characterized as the sum of several amplitude-modulated narrow frequency bands^6^. Using the Hilbert transform, the information contained within each band can be separated into rapidly varying temporal fine structure (TFS) and more slowly varying temporal envelope (ENV)^7,8^. From a signal-processing point of view, TFS can be described as the “carrier” signal while ENV corresponds to an amplitude modulator applied to the carrier. Previous studies have evaluated the role of TFS and ENV cues for speech perception^6–10^. To understand the contribution of each cue, some work has utilized acoustic chimaeras, stimuli which artificially merge the TFS from one sound with the ENV information of another sound^8^. In these studies, results show that, while either cue is sufficient for speech perception in quiet, both TFS and ENV are necessary when speech is embedded in background noise^7,9–11^. ENV speech cues, investigated by amplitude-modulating a noise carrier using a speech signal’s ENV, produce increased speech intelligibility as the number of frequency bands grow (e.g.^8^). Only 4 – 6 frequency bands are required for good speech intelligibility in quiet listening conditions^8,12^. In contrast, for TFS speech cues, investigated by amplitude-modulating a speech TFS carrier using the ENV of a noise token, an increase in the number of frequency bands decreases speech intelligibility^8^. While this describes an important dichotomy between ENV and TFS cues^6,8,13,14^, a body of work has shown that their independent impacts are challenging to evaluate, because the signal ENV can be reconstructed at the output of the auditory filters, even when it has been physically removed in the processing of a TFS speech stimulus^15–18^. An analysis of this effect for the stimuli used in the current study can be found in Supplementary Fig. S1.

Detecting sound motion is a crucial everyday task and has been studied extensively. For listeners with normal hearing (NH), the minimum angle that a sound has to move in order to be distinguished from a stationary sound is between 2° and 10°, depending on the signal duration, velocity, bandwidth and testing method (for a recent review, see^19^). Sound sources that move in space effectively traverse an array of multiple adjacent spatial locations, thereby resulting in rapidly changing binaural cues, namely interaural time and interaural level difference (ITD and ILD, respectively) cues. It has been suggested that sound motion detection is facilitated by a combination of displacement and velocity cues^20^, and ILDs have been shown to be more salient for sound velocity perception^21–23^. Consistent with that point, Warnecke and colleagues^24^ recently argued tracking level changes in the stimulus ENV as a moving sound traverses adjacent locations in space may aid listeners in identifying sound motion. However, they noted that sound motion detection was impeded when the signal ENV was slowly fluctuating, such as for speech stimuli, compared to when it was quickly fluctuating, such as for broadband noise stimuli. That study utilized single-word speech stimuli and spectrally-matched noise (SMN) tokens to create acoustic chimaeras composed of 8 frequency bands. This presented NH listeners with stimuli containing speech in the ENV and SMN in the TFS, or vice versa. Stimuli were either stationary or moving, and results showed that listeners were biased to report acoustic chimaeras with a slowly fluctuating speech-like ENV as stationary, while chimaeras with a quickly fluctuating SMN ENV did not show that bias. Notably, chimaeras with a speech-like ENV also had a higher speech intelligibility score than those with a SMN ENV. Those results demonstrate two confounding factors that may have been driving listeners’ bias to categorize sounds as stationary. Thus, it remains unclear whether the degree of short-term level fluctuation in the chimaera ENV, the intelligibility of chimaera content, or a combination of the ENV level and intelligibility induced a perceptual bias. Most importantly, those findings suggest that, under some stimulus conditions, sound motion processing and speech processing may compete for sound processing resources. Here, we investigate how sound motion perception is impacted when the target stimulus is an ecologically relevant stimulus such as speech, opening up the question of whether sound motion detection could also be modulated by auditory attention.

Previous studies describe some aspects of the functional importance of sound motion perception in NH adults^19^. However, what remains largely unknown to what extent signal ENV or speech intelligibility may modulate sound motion detection. The present study aimed to evaluate the impacts of signal ENV and speech intelligibility on sound motion perception. Acoustic chimaera stimuli are ideal for examining this question as they vary in the extent to which stimulus content is intelligibile^8,24^. We implemented Smith et al.’s approach^8^ for generating acoustic chimaeras to manipulate signal ENV and speech intelligibility. Given the recent findings by Warnecke and colleagues^24^, we predicted that sound motion perception is influenced by the degree to which listeners understand the content of a sound. Specifically, we hypothesized that when the stimulus content becomes more intelligible, listeners would exhibit increased bias towads perceiving sounds as stationary, because their attention may be momentarily drawn towards the content of the stimulus. Alternatively, a stationary bias could be governed by stimulus ENV, independently of the intelligibility of stimulus content, or a combination of these two components.

## Results

In this study, we tested the relative impacts of signal ENV (noise vs. speech) and intelligibility of the signal on sound motion perception. To do so, we created acoustic chimaeras from speech and SMN tokens (for details, see “Methods”). The full stimulus set entailed seven conditions with a speech-like ENV and seven conditions with a SMN ENV. For each ENV type, there were five chimaera stimuli created with 2, 4, 6, 8 and 16 frequency bands and two control stimuli. Chimaera stimuli that contained a speech-like ENV are referred to as Speech Chimaera (SC), while chimaera stimuli that contained a noise-like ENV are referred to as Noise Chimaera (NC). Sublettering indicates the frequency band manipulations within each SC and NC (SC_2_, SC_4_, SC_6_, SC_8_, SC_16_; NC_2_, NC_4_, NC_6_, NC_8_, NC_16_). Two stimulus types were added as control: (1) to test whether sound motion perception is affected differently for chimaera and non-chimaera stimuli, we added the original stimuli for each ENV type (original speech: OR_S,_ original SMN: OR_SMN_); (2) to test whether sound motion perception is affected differently when the ENV type remains, but the stimulus content becomes unintelligible, we added a reversed 16-band chimaera for each ENV type (reversed speech: R_S,_ reversed SMN: R_SMN_). In a sound-proofed chamber, each stimulus condition was presented from a frontal position both as stationary and left- or rightward moving a 10° angular range. After sound presentation, NH listeners reported their perceived sound motion in a one alternative forced-choice task, and verbally repeated what they understood.

### Speech intelligibility

Speech intelligibility was measured as the ratio of the number of stimuli whose content was correctly understood to the number of all presented stimuli.

**Figure 1:**
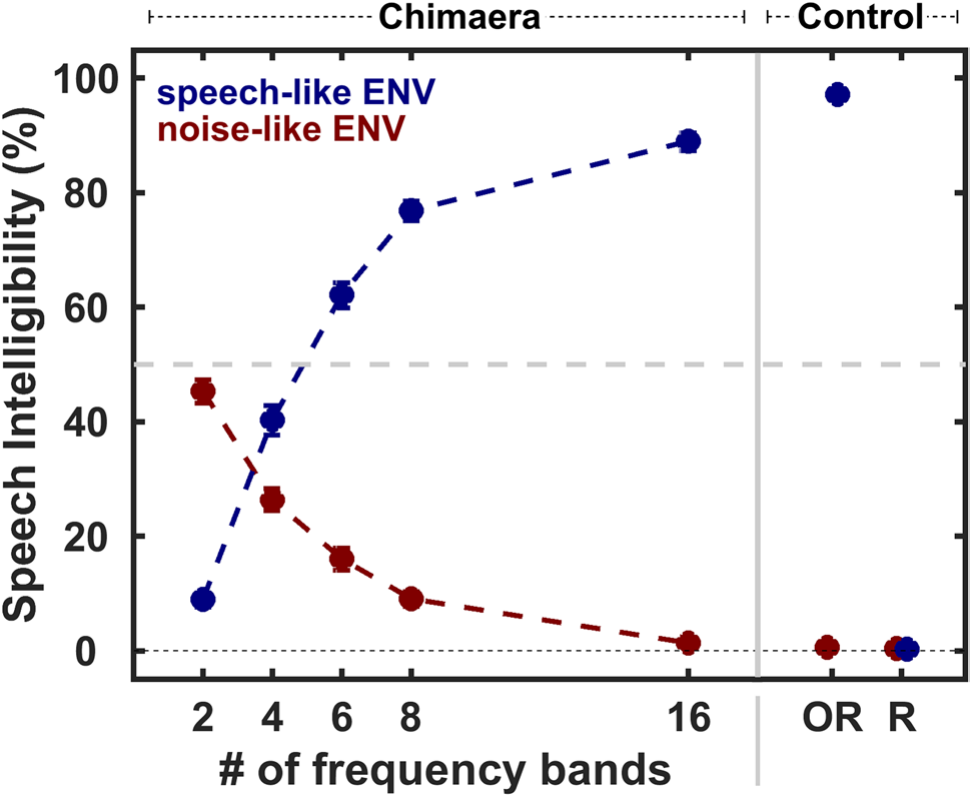
Speech intelligibility across conditions for each ENV type. Speech intelligibility (mean ± sem) was measured as a function of chimaera and control conditions, with stimulus ENV type indicated by color. As the number of frequency bands increased, intelligibility of sound content increased for chimaera with a speech-like ENV (blue), but decreased for chimaera with a noise-like ENV (red). During control conditions, listener’s performance on sound content intelligibility reached ceiling levels for original speech (OR_S_), but floor levels for SMN (OR_SMN_) and both reversed control stimuli (R_S_/R_SMN_).

Figure 1 plots the mean speech intelligibility score (± standard error of the mean, sem) as a function of condition for each ENV type (color legend), along the five chimaera conditions and both control conditions. Overall, speech intelligibility followed the expected trend for both SC (blue) and NC (red) stimuli as previously shown^8^. As the number of frequency bands increased, speech intelligibility increased for SC conditions, from less than 10% (SC_2_) to 89% (SC_16_), but decreased for NC conditions, from 45% (NC_2_) to 0% (NC_16_). Speech intelligibility scores in the present study were generally lower than those reported by Smith and colleagues^8^. However, this is to be expected, as we tested performance for single words, while Smith et al. (2002) utilized whole sentences, which provided more semantic context that is known to aid recognition. As expected, speech intelligibility was best for OR_S_, reaching ceiling performance at 100%, and poorest for OR_SMN_ and both reversed stimuli, R_S_ and R_SMN_, which showed floor performance at 0%.

### Sensitivity to sound motion

**Figure 2:**
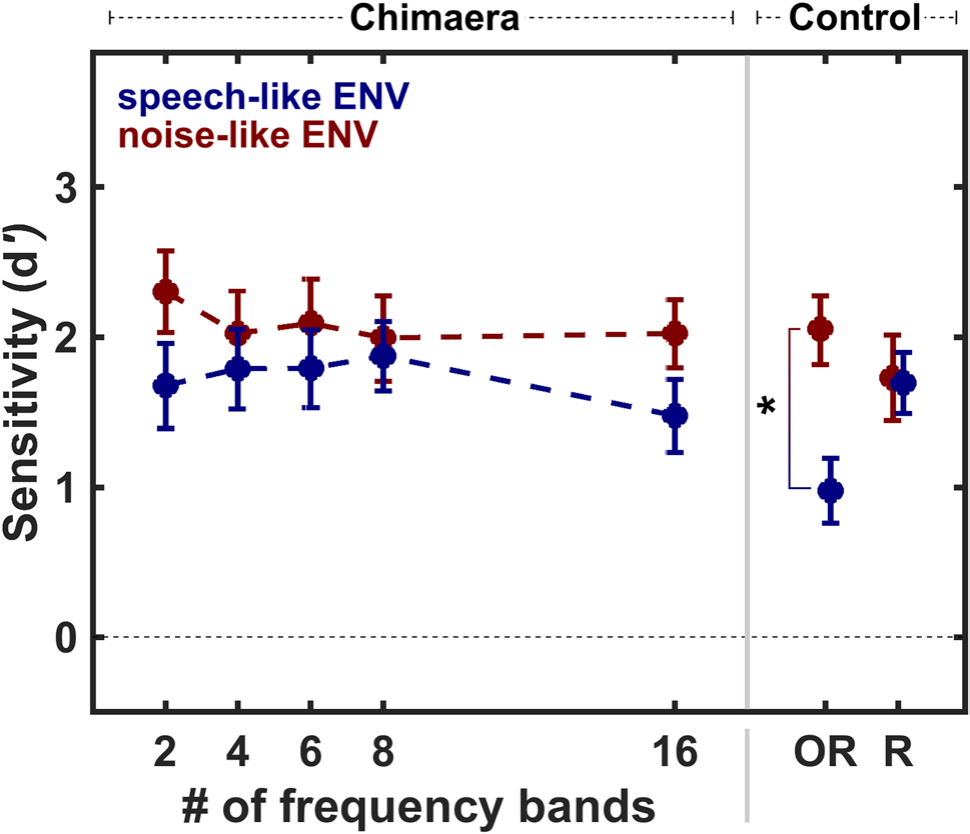
Sensitivity to sound motion across conditions for each ENV type. Sensitivity scores (*d’*; mean ± sem) are plotted as a function of chimaera and control conditions, with ENV type indicated by color. Higher scores indicate better discriminability. Across chimaera conditions, listeners were significantly more sensitive to sound motion for stimuli with noise-like ENVs (red) compared to speech-like ENVs (blue). In control conditions, original stimuli did not differ from chimaera stimuli, but listeners were significantly less sensitive to sound motion when stimuli were speech (OR_S_) compared to SMN (OR_SMN_).

Recent work by Warnecke and colleagues showed no effect of sensitivity to sound motion between 8-band acoustic chimaeras with speech- or noise-like ENVs^24^. However, the results of that study suggest that the listeners have better sensitivity to sound motion for unprocessed stimuli and little to no response bias for all unprocessed and chimaera stimuli with a noise-like ENV. This led us to predict that listeners would show better sensitivity to sound motion for unprocessed stimuli with noise-like ENVs compared to speech-like ENVs. To evaluate how well listeners could distinguish between stationary and moving stimuli, we calculated sensitivity (*d’*) for each condition. Figure 2 illustrates listeners’ mean sensitivity to sound motion (± sem) across conditions, where a greater *d’* value indicates better sensitivity for sound motion. To evaluate the impact of signal ENV on how well listeners could distinguish between stationary and moving sounds, we analyzed the chimaera conditions using a 5 × 2 repeated measures analysis of variance (ANOVA). The number of frequency bands (2, 4, 6, 8, 16) and ENV type (noise/speech) were entered as within-subject factors. As expected, we found a significant main effect for ENV type (F_1,21_ = 11.27, p = 0.003), indicating that listeners were more sensitive to sound motion when presented with chimaera stimuli that had a noise-like ENV (mean = 2.08, sem = 0.12), compared to a speech-like ENV (mean = 1.72, sem = 0.11). There was no main effect for the number of frequency bands (F_4,84_ = 1.5, p = 0.2) and no interaction (F_4,84_ = 0.92, p = 0.45).

Our experiment contained two control stimulus types to evaluate (1) whether sensitivity to sound motion would differ between chimaeric stimuli (SC_16_/NC_16_) and non-chimaeric stimuli (OR_S_/OR_SMN_), and (2) whether sensitivity would be impacted when the speech intelligibility between chimaeric stimuli differed (SC_16_/NC_16_ vs. R_S_/R_SMN_). We tested the difference in listeners’ sensitivity to sound motion for each of these controls using factorial ANOVA analyses. First, a 2 × 2 ANOVA for ENV type (noise/speech) and stimulus type (Chimaera/Original) showed a main effect of ENV type (F_1,84_ = 12.44, p = 0.0007; noise: mean = 2.03, sem = 0.16; speech: mean = 1.22, sem = 0.16), but no effect of the stimulus type (F_1,84_ = 1.22, p = 0.30), and no interaction (F_1,84_ = 1.3, p = 0.25). These results indicate that ENV was a prominent cue for sensitivity to sound motion, independently of whether stimuli were modified to be chimaeric, or not. Further, in a second 2 × 2 ANOVA for ENV type (noise/speech) and stimulus type (Chimaera/Reversed Chimaera), we found no significant main effect or interaction (whole model test: F_3,84_ = 0.8, p = 0.45). This indicates that listeners’ sensitivity to sound motion was not substantially impacted by the intelligibility of a stimulus’ content; a finding that is further supported by the non-significant impact of number of frequency bands on sound motion sensitivity (see above).

Previous research indicates that there is no significant difference between localizing stationary speech and broadband signals, such as SMN^24–27^. However, it is unclear whether this trend extends to listener’s sensitivity for detecting the sound motion of speech and SMN signals. Sound motion sensitivity was evaluated for stimuli which represented the original speech (OR_S_) and broadband (OR_SMN_) signals. To do so, we used a two-tailed t-test between the OR_S_ and OR_SMN_ conditions, and found that listeners were significantly less sensitive to sound motion (t(42) = -3.36, p = 0.0016) when presented with OR_S_ stimuli (mean = 0.97, SEM = 0.21) as compared to OR_SMN_ (mean = 2.05, SEM = 0.23), indicating that it was more difficult to detect sound motion when stimuli were original speech compared to SMN.

### Response bias for sound motion

Studies on psychophysical phenomona commonly use *d’* indices of sensitivity as a primary measure of interest. However, a subject’s decision-making process can be biased. For example, an equally detectable signal can have the same percent correct performance, but opposite response biases in two subjects^28^. In order to fully assess performance, the response bias, also known as the decision criterion (*c*), is thus imperative. Previous research found that listeners showed a response bias for stimuli with speech-like ENVs, judging them as stationary compared to stimuli with noise-like ENVs, which did not show that bias^24^. However, the content of stimuli with speech-like ENVs was also perceived as intelligible speech more often compared to the content of stimuli with noise-like ENVs. This created a confound as to what was driving the stationary response bias.

One possibility is that the previously observed response bias was influenced exclusively by the signal ENV. In that case, independently of how well listeners understand the content of each stimulus, all stimuli with a speech-like ENV (SC_2-16_, OR_S_ and R_S_) should induce a stationary response bias, i.e. a bias criterion value less than 0. Moreover, independently of how well listeners understand the content of each stimulus, all stimuli with a noise-like ENV (NC_2-16_, OR_SMN_ and R_SMN_) should then show a non-stationary bias, i.e. a criterion value greater than or equal to 0. By contrast, if the response bias was influenced exclusively by how well listeners understood the content of each stimulus, i.e. its speech intelligibility, then an increase in intelligibility scores should induce an increase in response bias to report sounds as stationary, independently of the signal’s ENV (e.g. NC_2_, SC_4-16_, OR_S_). Importantly, if content intelligibility contributes to stationary bias, then removing only content intelligibility (R_S_) should remove the bias. Finally, it is possible that an interaction of speech intelligibility and ENV type promotes response biases to sound motion.

**Figure 3:**
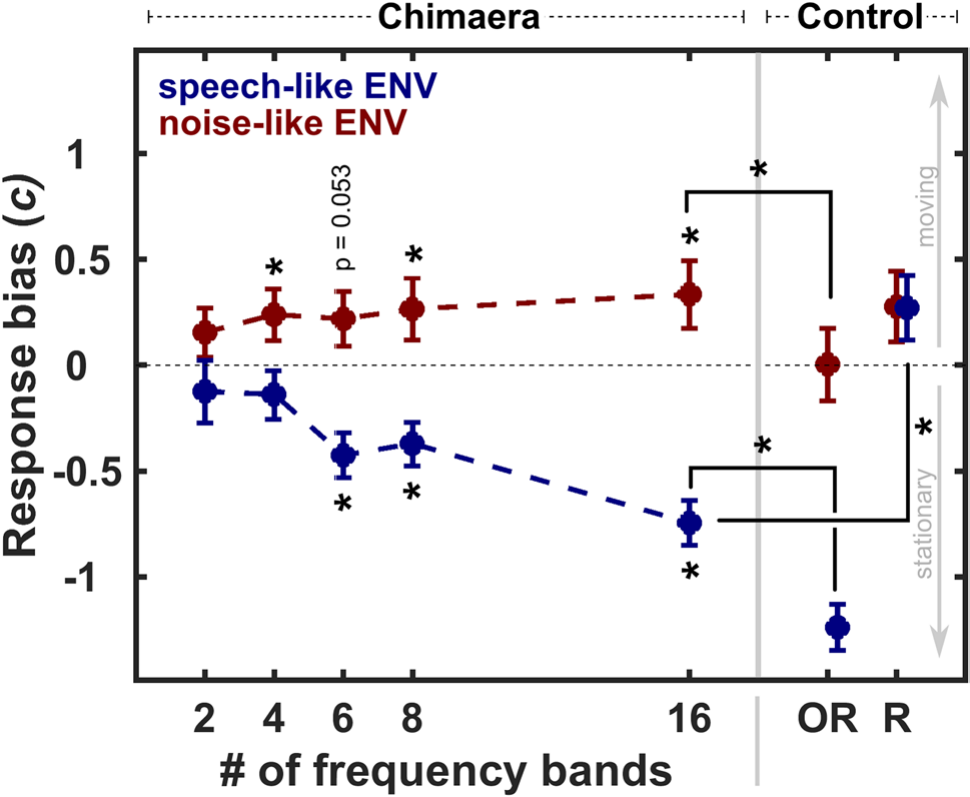
Response bias for sound motion across conditions for each ENV type. Response bias (*c*; mean ± sem) is plotted as a function of chimaera and control conditions, with ENV type indicated by color. Positive numbers indicate a bias towards judging sounds as moving, while negative numbers indicate a bias towards judging sounds as stationary (grey arrows). Statistical significance (see “Results”) is indicated by stars. Listeners showed a stationary perceptual bias for chimaera stimuli that had a speech-like ENV (blue) and at least six frequency bands. By contrast, chimaera stimuli with a noise-like ENV (red) and 4, 8 or 16 frequency bands induced a moving perceptual bias. Bias differed significantly for control conditions, in which listeners showed no bias for OR_SMN_, while the strongest stationary bias was induced for OR_S_. Removing sound content intelligibility, R_S_, removed the stationary bias that was observed in listeners when the same sound’s content was intelligible, SC_16_.

Figure 3 plots listeners’ mean response bias criterion (± sem) across conditions. While a criterion of 0 indicates no response bias, bias criteria greater than 0 or less than 0 indicate a bias towards judging sounds as moving, or stationary, respectively. Perfect scores were adjusted by the ratio of 1 to the doubling of hits and misses. Previous work informed apriori predictions for the response biases of SC and NC stimuli (see above), leading us to evaluate whether signal ENV biased listeners towards judging sounds as stationary or moving using a one-tailed t-tests for each chimaera condition. As we tested simple effects for each condition, we did not adjust our alpha and tested at α = 0.05. The details of statistical testing can be found in Table 1.

**Table 1:** Response Bias statistical analyses. For each tested condition (first column), we provide details of the statistical analyses (t-value, p-value, sem, Range) for chimaera and control conditions with noise-like (left main column) and speech-like (right main column) ENVs.

# Chs	Noise-like ENVs				Speech-like ENVs			
	t	Prob > t	sem	Range	t	Prob < t	sem	Range
2	1.332	0.090	0.116	2.164	−0.835	0.206	0.149	3.172
4	1.958	0.032	0.122	2.839	−1.203	0.121	0.115	2.545
6	1.693	0.053	0.130	2.624	−3.988	0.0003	0.107	2.020
8	1.834	0.040	0.144	2.438	−3.574	0.0009	0.104	1.814
16	2.112	0.023	0.158	2.536	−7.112	< 0.0001	0.105	1.668
OR	0.030	0.488	0.171	3.172	−11.330	< 0.0001	0.109	1.395
R	1.678	0.054	0.165	2.793	1.801	0.957	0.151	2.711

All conditions with stimuli containing a noise-like ENV (NC_2-16_, OR_SMN_, R_SMN_) had bias criteria equal to or greater than 0. When testing the NC conditions, all but one condition with four or more frequency bands were significantly larger than 0 (see Table 1; NC_4_: mean = 0.24, sem = 0.12; NC_8_: mean = 0.26, sem = 0.14; NC_16_: mean = 0.33, sem = 0.16). NC_6_ approached significance (Table 1; mean = 0.22, sem = 0.13), indicating that these conditions showed a small bias for being judged as moving. Analogously, conditions with a speech-like ENV (SC_2-16_, OR_S_) except R_S_, had bias criteria less than 0, and all SC stimuli with at least six frequency bands were significantly smaller than 0 (see Table 1; SC_6_: mean = -0.42, sem = 0.1; SC_8_: mean = -0.37, sem = 0.1; SC_16_: mean = -0.74, sem = 0.1). This confirms that listeners were substantially biased towards judging SCs with at least six frequency bands as stationary.

We evaluated whether there was a difference in response bias for our control conditions to understand the impact of (1) chimaeric vs non-chimaeric stimulus types and (2) speech intelligibility. First, a 2 × 2 ANOVA for ENV type (noise/speech) and stimulus type (Chimaera/Original) revealed main effects for both variables (ENV type: F_1,84_ = 69.78, p < 0.0001; stimulus type: F_1,84_ = 8.73, p = 0.0041) and no significant interaction (F_1,84_ = 0.34, p = 0.55). As such, stimuli with a speech-like ENV had an overall smaller response bias value than stimuli with a noise-like ENV, mirroring the data distribution of all chimaera conditions. Further, stimuli of both original conditions (OR_S_/OR_SMN_) had a smaller response bias value compared to stimuli of both chimaeric conditions (SC_16_/NC_16_), which effectively removed response bias for the broadband signal (OR_SMN_, mean = 0, sem = 0.17), and revealed the strongest response bias towards a stationary percept for the speech signal (OR_S_, mean = -1.23, sem = 0.11; Fig. 3). These findings corroborate results from Warnecke and colleagues^24^. Second, to test whether the intelligibility of a stimulus’ content impacted response bias, a 2 × 2 ANOVA for ENV type (noise/speech) and stimulus type (Chimaera/Reversed Chimaera) showed that both main effects and their interaction were significant (ENV type: F_1,84_ = 13.66, p = 0.0004; Chimaera/Reversed Chimaera: F_1,84_ = 10.7, p = 0.0016; interaction: F_1,84_ = 13.42, p = 0.0004). This indicates that reversing the stimulus in an effort to remove the intelligibility of its content substantially removed listeners’ response bias. Importantly, post-hoc comparisons using Tukey’s HSD at α = 0.05 indicated that SC_16_ differed significantly from all other control conditions (NC_16_, R_S_, R_SMN_). This interaction highlights that the decrease in response bias for this control condition was driven by the difference between the two chimaera conditions with a speech-like ENV that manipulated intelligibility of stimulus content (SC_16_ vs. R_S_). Further, R_S_, the temporally-reversed SC_16_, which retained the speech-like ENV but had no intelligible content (R_S_, Fig. 1), was not significantly different from 0 in a subsequent two-tailed t-test to evaluate its response bias (t(21) = 1.8, p = 0.086; mean = 0.27, sem = 0.15). This indicates that there was no considerable response bias for this stimulus.

Collectively, our results indicate that sound motion perception is impacted by an interaction of ENV type and speech intelligibility, because substantial stationary bias is (1) not observed for all stimuli with a speech-like ENV (e.g. SC_2_, SC_4_, R_S_), and (2) not observed for all stimuli whose content is at least in part intelligible to listeners (e.g. NC_2_). Instead, noise-like ENVs induced either no bias or a small bias toward a moving percept, while speech-like ENVs induce either no bias or a strong bias toward a stationary percept. Importantly, only for stimuli with speech-like ENVs, increasing stimulus content intelligibility increased stationary bias (e.g. SC_6_, SC_8_, SC_16_, OR_S_,), and removing content intelligibility removed stationary bias for sound motion detection (e.g. SC_16_ vs. R_S_).

## Discussion

In real-life listening situations we are confronted with a cacophony of sounds, which can be stationary but are often in motion relative to a listener’s head. Spatial auditory perception provides awareness for our surroundings. Crucially, it serves to localize sounds, categorize them as useful or dangerous, and enhance communication by selectively guiding attentional resources to a talker or location of interest^2,29,30^.

Under ideal conditions, humans can localize stationary broadband sounds with impressive acuity^31–35^. The ability to localize sounds in space depends on the integration of several binaural acoustic cues, namely ITDs and ILDs^31,34^. A few studies have shown that listeners are able to localize stationary speech sounds in the horizontal plane as accurately as non-speech broadband stimuli^26,27,36^, and recent work showed that there is no difference in localization accuracy for a stationary acoustic chimaera with speech-like or noise-like ENVs^24^. Extending these findings of stationary sound localization, we investigated listeners’ sensitivity to sound motion. While we found no differences in motion sensivity as the number of frequency bands (i.e. stimulus conent intelligibility) changed, results showed that sound motion was overall more difficult to detect for stimuli with speech-like ENVs compared to stimuli with noise-like ENVs (Fig. 2). Further, we found a significant difference in sound motion sensitivity for the original speech stimulus (OR_S_) compared to the original noise stimulus (OR_SMN_), two stimuli which show no difference in their stationary localizability^24–27^. Furture work on this result would benefit from further controlling the effective speech duration to more aptly compare these two types of stimuli. Together, the sensitivity results point to an important difference in the ability to locate a sound vs. detect its motion, suggesting that different underlying mechanisms could contribute to sound motion detection.

Moving sounds create dynamic binaural changes in ITDs and ILDs. Previous work suggests that dynamic ILD cues may be more salient than ITD cues to discriminate sound velocity^21–23,31^, an important component of auditory motion perception^20^. We recently proposed that listeners may be tracking changes in the short-term level of a moving signal across spatial locations^24^. That is, when a moving acoustic signal contains a quickly-changing noise-like ENV, a listener would be comparing approximately equal energy levels from one spatial location to the next, indicating that perceived changes in ILDs are mostly due to changes in spatial location. This may help the listener to detect whether a sound moved. In support of that hypothesis, the present study found that listeners were more sensitive to sound motion for any stimuli that contained a noise-like ENV (Fig. 2). In contrast, when a moving acoustic signal contains a speech-like ENV, a listener would be comparing energy levels that vary more slowly in their short-term level across time, while they also move from one spatial location to the next. In such a case, when the sound is in motion, ILD changes could be due to changes in spatial location or changes in the temporal short-term level from the signal itself. These co-occurring cues may confound the judging of sound motion, leading listeners to perceive sounds with a speech-like ENV as stationary.

This conjecture suggests that sound ENV contributes to sound motion detection. However, the results of the present study indicate that not all stimuli with a speech-like ENV result in a stationary bias (e.g. SC_2_, SC_4_, R_S_). Hence, at least one other factor may influence sound motion detection.

Stationary response bias increased when listener’s understanding of the content of the stimulus increased, indicating that speech intelligibility may impact sound motion detection when coupled with a speech-like ENV (Fig. 3). Increasing the number of frequency bands of the SC effectively also increased their spectro-temporal resolution, improving the stimulus content intelligibility (Fig. 1). As the intelligibility of stimulus content increased, so did the stationary response bias, reaching statistical significiance for any SC with at least 6 frequency bands (Fig. 3). Comparing the SC with highest content intelligibility, SC_16_, to its reversed version, R_S_, provides strong evidence that speech intelligibility is a salient component of the stationary response bias for sound motion, because reversing SC_16_ retained all temporal and spectral components of the chimaera, but removed the intelligibility of its content (Fig. 1). An alternative control in future work could involve foreign word stimuli to exclude the possibility that differences in speech onset and decay time contributed to this effect^37^. In combination with the systematic increase in SC stimuli being categorized as stationary when their content intelligibility improved (Fig. 3), the results demonstrate that sound content intelligibility is one contributor to stationary biases for moving sounds. Note that sounds were not biased to be perceived as stationary in the NC_2_ condition, where stimuli had reached about 45% intelligibility. It is possible that independently of stimulus ENV, a stationary bias for a moving sound may only manifest itself when there is a certain minimum stimulus content intelligibility (for example, stationary bias for SC stimuli was only observed when stimulus content intelligibility was at least 62%).

Collectively, our results show that sensitivity to sound motion is accurate for the SMN stimulus, as evident by listeners’ high sensitivity scores in that condition (OR_SMN_, Fig. 2) and the absence of a response bias (OR_SMN_, Fig. 3). Further, and importantly, sound motion perception in the auditory system appears biased: acoustic chimaera stimuli with a noise-like ENV induce a small moving bias, while both chimaeric and non-chiameric stimuli with a speech-like ENV induce a stationary bias. Importantly, increasing the content intelligibility of stimuli with a speech-like ENV systematically increases stationary bias (Fig. 3), and presenting listeners with clear speech induces the strongest bias toward a stationary percept (OR_S_, Fig. 3). Further, removing intelligible content from acoustic stimuli removes the stationary perceptual bias (R_S_, Fig. 3).

Previous work suggested that the processing of speech is similar to that of non-speech stimuli^1^, but it has since been argued that speech might be treated differently than non-speech sounds in auditory perception^38,39^. In fact, our results show that with regard to sound motion perception, the two types of stimuli show different perceptual biases. Recent neuroimaging studies demonstrated distinct neural systems in the auditory processing of intelligible compared to unintelligible speech (e.g.^40,41^), giving evidence for an anterior auditory “what” pathway. Subsequent work demonstrated differential activation of the auditory “where” and “what” pathways when listeners were asked to attend to sound location or feature^42^, outlining parallel processing for sounds in the auditory “where” and “what” pathways. Using magnetoencephalographic imaging, researchers determined the time scale of pathway activation and showed that a dissociation between the “where” and “what” pathways started after about 100 ms. Importantly, the “where” pathway was activated about 30 ms earlier than the “what” pathway. In the present study, a dual-task was implemented whereby listeners were asked to report sound motion – thereby needing to localize sounds – and the content of the sound. It is likely that the processing of this task engaged the auditory “where” and “what” pathways. Assuming that the activation of the “where” pathway occurred about 30 ms earlier than that of the “what” pathway, determining sound location could have preceded processing of sound content. Evidence from speech recognition studies has demonstrated that incoming information, such as natural speech, is segmented and processed on the basis of a sliding window of temporal integration. In the context of speech, psychophysical research and theoretical work suggest that two temporal windows may be sliding in parallel in the temporal domain, processing the acoustic signals at the syllabic (~ 125–200 ms) and the phonemic level (~ 25–50 ms)^43–45^. If, after initial determination of sound location, the sound content was perceived to be intelligible, subsequent speech processing of our disyllabic stimuli may have prevented the detection of simultaneously-occurring changes in sound location of the moving sound, thereby facilitating the stationary percept of these stimuli.

Additional reinforcement of the perceptual bias for the processing of moving speech may have been driven by selective attention: Human neurophysiological work has revealed that selectively attending to phonetic content increases the neural response in the “what” pathway, while selective attending to sound location increases the neural response in the “where” pathway^42^. This could reflect selectivity for task-relevant information. In the present study, listeners were not told to attend to either sound location or content; rather, they performed a dual-task. If a listener’s attention was momentarily guided toward the content of acoustic stimuli which contained linguistically-relevant content, it is possible that this selective attention contributed to processing of the sound content at the expense of processing its motion.

Our work shows that listeners are biased in processing acoustic information, indicating that the auditory system can be mislead. In understanding the system’s bias, we can utilize the knowledge of misperceptions to guide studies on auditory processing and representations, improve processing algorithms for devices that aid listeners with hearing impairments, and refine the creation of virtual acoustic environments.

## Methods

### Participants

Twenty-two listeners (ages 19 to 24 years, avg. 20 years) participated in this study, and received either university credit or payment for their participation. All listeners passed hearing screening at octave frequencies between 250 and 8000 Hz, defined as thresholds ≤ 20 dB HL, and none had extensive experience as research participants in psychoacoustic studies. All participants were naïve to the study’s experimental design and purpose and gave written informed consent prior to experiment. All experimental procedures followed the regulations set by the National Institutes of Health and were approved by the University of Wisconsin’s Health Sciences Institutional Review Board.

### Test stimuli and experimental design

In the experiment described below, speech tokens were 420 unique disyllabic words from the Isolated Words corpus^46^, spoken by multiple male and female talkers and recorded at 44.1 kHz. All words started with a consonant. The collection of words had an average duration of 515 ms, ranging from 381 to 656 ms.

To create variations of individual speech tokens, we followed several steps of stimulus creation. First, a matching SMN was created for each word by synthesizing noise of the same power spectrum and duration of each speech token, via randomizing the phase of its Fourier spectrum. Second, to create chimaeras of the speech and SMN signals, we utilized Smith et al.’s (2002) Chimaera-generating approach^8^. The original speech token (OR_S_) and SMN stimulus (OR_SMN_) were each bandpass-filtered into 2, 4, 6, 8 and 16 frequency bands between 200 to 8000 Hz according to the Greenwood function^47^. High-frequency content (> 8 kHz) is needed for accurate localization in the vertical, polar plane, but not the horizontal, medial plane^48^, which we tested here. Subsequently, for each frequency band, the ENV and TFS of both the OR_S_ and OR_SMN_ signals were extracted using Hilbert transform and exchanged, such that the ENV of one signal was superimposed on the TFS of the other, and vice versa. The newly created signals were summed in the time domain to form a multi-band chimaera. As such, for each pair of a speech token, OR_S_, and its matching SMN, OR_SMN_, and for each number of frequency bands, two chimaeras were created: one containing speech-like ENV and the other containing SMN ENV. For this study, speech chimaera (SC) refers to chimaera stimuli with a speech-like ENV and SMN TFS, whereas noise chimaera (NC) refers to chimaera stimuli with a SMN ENV and speech TFS. Third, to test the impact of envelope independently of speech intelligibility, a set of acoustic stimuli were created by reversing the 16-band SC (R_S_) and the 16-band NC (R_SMN_) stimulus in the time domain. In total, the tested conditions contained 7 stimuli with a speech-like ENV (SC_2_, SC_4_, SC_6_, SC_8_ SC_16_, OR_S_, R_S_), as well as 7 stimuli with a SMN ENV (NC_2_, NC_4_, NC_6_, NC_8_, NC_16_, OR_SMN_, R_SMN_). A total of 5,880 stimuli were created from these fourteen conditions for each of the 420 disyllabic words. Each of the fourteen conditions was presented 15 times as stationary, and 15 times as moving, totaling 420 trials per participant. All stimuli and sound motions (stationary/moving) were pseudo-randomly assigned. All sounds started at 0º, and while stationary sounds remained there for their duration, moving sounds traveled a 10º angular distance, left- or rightward, and always ended at the ± 10° azimuthal ranges. We calculated sensitivity and response bias as a measure of sound motion identification for each condition.

### Apparatus

All testing was done in a sound booth (internal dimensions: 2.9 m × 2.74 m × 2.44 m; Acoustic Systems, Austin, TX, USA) covered in acoustic foam on the walls and ceiling (Pinta Acoustics, Minneapolis, MN, USA). Participants sat in a chair in the middle of a horizontal 37-loudspeaker array (Cambridge SoundWorks, North Andover, MA, USA; TDT Technologies, Alachua, FL, USA), which spanned azimuthal locations from—90° (left) to + 90° (right) in 5° resolution. The loudspeaker array was hidden behind a black curtain that was acoustically transparent to remove visual cues from the loudspeakers. Vector-base amplitude panning (VBAP) was implemented to create a continuously moving sound source along a trajectory, by panning between groups of adjoining loudspeakers^49^. Time-domain inverse filters for each loudspeaker were implemented to correct for flat frequency responses at the loudspeaker output. For all moving sounds, the gain coefficients for motion panning between the two adjoining loudspeakers together were adjusted to an output level normalized to 65 dBA, ensuring smooth panning. For all stationary sounds, the gain was calibrated to be 65 dBA SPL at the location of the participant’s head using a sound level meter (System 824, Larson Davis, Depew, NY, USA). A small touchscreen (34 cm, 13.3 inch diagonal; OnLap 1303, GeChic, Taichung City 403, Taiwan) was provided to the participant to start a trial and provide responses during the task.

### Procedure

Prior to the main experiment, participants were familiarized with the experimental stimuli by listening to 28 randomly selected examples of stimuli that represented each of the fourteen conditions, once as stationary, and once as moving. These 28 stimuli were not part of a participant’s stimulus set for the main experiment. During familiarization, participants were not told that the stimuli would be stationary or moving and the only task was to repeat what they understood.

During the main experiment, listeners were asked to identify both the motion of the sound (i.e., classify sound as “stationary” or “moving”) and the content of the sound. As such, the experiment employed a dual-task utilizing a one alternative forced choice testing paradigm. On a given trial, participants pressed the touchscreen to start a trial. Subsequently, a sound (stationary or moving) played from the central location of the horizontal loudspeaker array. Participants were instructed to face forward and keep their head still during sound presentation, and after sound offset they could move their head. After sound offset, the frontal touch screen displayed the sentence “The sound I just heard was …” and two selection boxes labeled “stationary” and “moving.” Participants indicated their perceived sound motion by selecting a choice. To indicate the perceived sound content, participants verbally repeated what they understood, which the experimenter, who was seated outside of the testing chamber, manually recorded. Subsequently, participants could start a new trial by pressing the touchscreen.

## Supplementary Information

Supplementary Information.

## Data Availability

The datasets generated during and/or analysed during the current study are available from the corresponding author on reasonable request.

## References

[1] Bregman AS (1990). Auditory Scene Analysis: The perceptual organization of sound.

[2] Kidd G, Arbogast TL, Mason CR, Gallun FJ (2005). The advantage of knowing where to listen. J. Acoust. Soc. Am..

[3] Freyman RL, Helfer KS, McCall DD, Clifton RK (1999). The role of perceived spatial separation in the unmasking of speech. J. Acoust. Soc. Am..

[4] Kreitewolf J, Lewald J, Getzmann S (2011). Effect of attention on cortical processing of sound motion: an EEG study. Neuroimage.

[5] Getzmann S, Lewald J (2011). The effect of spatial adaptation on auditory motion processing. Hear. Res..

[6] Flanagan JL (1980). Parametric coding of speech spectra. J. Acoust. Soc. Am..

[7] Lorenzi C, Moore BCJ (2007). Role of temporal envelope and fine structure cues in speech perception: A review. Proceedings of the International Symposium on Auditory and Audiological Research.

[8] Smith ZM, Delgutte B, Oxenham AJ (2002). Chimaeric sounds reveal dichotomies in auditory perception. Nature.

[9] Sheft S, Ardoint M, Lorenzi C (2008). Speech identification based on temporal fine structure cues. J. Acoust. Soc. Am..

[10] Lorenzi C, Gilbert G, Carn H, Garnier S, Moore BCJ (2006). Speech perception problems of the hearing impaired reflect inability to use temporal fine structure. Proc. Natl. Acad. Sci..

[11] Moore BCJ (2008). The role of temporal fine structure processing in pitch perception, masking, and speech perception for normal-hearing and hearing-impaired people. J. Assoc. Res. Otolaryngol..

[12] Shannon RV, Zeng F-G, Kamath V, Wygonski J, Ekelid M (1995). Speech recognition with primarily temporal cues. Science (80-).

[13] Zeng F-G (2004). On the dichotomy in auditory perception between temporal envelope and fine structure cues (L). J. Acoust. Soc. Am..

[14] Drullman R (1995). Temporal envelope and fine structure cues for speech intelligibility. J. Acoust. Soc. Am..

[15] Gilbert G, Lorenzi C (2006). The ability of listeners to use recovered envelope cues from speech fine structure. J. Acoust. Soc. Am..

[16] Ghitza O (2001). On the upper cutoff frequency of the auditory critical-band envelope detectors in the context of speech perception. J. Acoust. Soc. Am..

[17] Shamma S, Lorenzi C (2013). On the balance of envelope and temporal fine structure in the encoding of speech in the early auditory system. J. Acoust. Soc. Am..

[18] Swaminathan J, Reed CM, Desloge JG, Braida LD, Delhorne LA (2014). Consonant identification using temporal fine structure and recovered envelope cues. J. Acoust. Soc. Am..

[19] Carlile S, Leung J (2016). The perception of auditory motion. Trends Hear..

[20] Shayman CS (2020). Frequency-dependent integration of auditory and vestibular cues for self-motion perception. J. Neurophysiol..

[21] Altman JA, Romanov VP, Pavlov IP (1988). Psychophysical characteristics of the auditory image movement perception during dichotic stimulation. Int. J. Neurosci..

[22] Grantham DW (1984). Discrimination of dynamic interaural intensity differences. J. Acoust. Soc. Am..

[23] Carlile S, Best V (2002). Discrimination of sound source velocity in human listeners. J. Acoust. Soc. Am..

[24] Warnecke M, Peng ZE, Litovsky RY (2020). The impact of temporal fine structure and signal envelope on auditory motion perception. PLoS ONE.

[25] Grantham DW, Ashmead DH, Ricketts TA, Labadie RF, Haynes DS (2007). Horizontal-plane localization of noise and speech signals by postlingually deafened adults fitted with bilateral cochlear implants. Ear Hear..

[26] Begault DR, Wenzel EM (1993). Headphone localization of speech. Hum. Factors.

[27] Ricard GL, Meirs SL (1994). Intelligibility and localization of speech from virtual directions. Hum. Factors.

[28] Azzopardi P, Cowey A (1998). Blindsight and visual awareness. Conscious. Cogn..

[29] Kopčo N, Best V, Carlile S (2010). Speech localization in a multitalker mixture. J. Acoust. Soc. Am..

[30] Shinn-Cunningham BG, Ihlefeld A, Larson E (2005). Bottom-up and top-down influences on spatial unmasking. Acta Acust. United with Acust..

[31] Blauert J (1997). Spatial hearing: the psychophysics of human sound localization.

[32] Carlile, S. The physical and psychophysical basis of sound localization. in *Virtual auditory space: Generation and applications* 27–78. (Springer, 1996).

[33] Litovsky RY, Macmillan NA (1994). Sound localization precision under conditions of the precedence effect: effects of azimuth and standard stimuli. J. Acoust. Soc. Am..

[34] Middlebrooks JC, Green DM (1991). Sound localization by human listeners. Annu. Rev. Psychol..

[35] Perrott DR (1984). Concurrent minimum audible angle: A re-examination of the concept of auditory spatial acuity. J. Acoust. Soc. Am..

[36] Carlile S, Leong P, Hyams S, Pralong D (1997). The nature and distribution of errors in the localization of sounds by humans. Hear. Res..

[37] Irino T, Patterson RD (1996). Temporal asymmetry in the auditory system. J. Acoust. Soc. Am..

[38] Liberman AM (1996). Speech: A special code.

[39] Liberman, A. M. On finding that speech is special. in *Handbook of Cognitive Neuroscience* 169–197 (Springer, 1984).

[40] Scott SK, Blank CC, Rosen S, Wise RJS (2000). Identification of a pathway for intelligible speech in the left temporal lobe. Brain.

[41] Okada K (2010). Hierarchical organization of human auditory cortex: evidence from acoustic invariance in the response to intelligible speech. Cereb. Cortex.

[42] Ahveninen J (2006). Task-modulated “what” and “where” pathways in human auditory cortex. Proc. Natl. Acad. Sci..

[43] Luo H, Poeppel D (2007). Phase patterns of neuronal responses reliably discriminate speech in human auditory cortex. Neuron.

[44] Luo H, Poeppel D (2012). Cortical oscillations in auditory perception and speech: evidence for two temporal windows in human auditory cortex. Front. Psychol..

[45] Poeppel D (2003). The analysis of speech in different temporal integration windows: cerebral lateralization as ‘asymmetric sampling in time’. Speech Commun..

[46] Brouwer S, Bradlow AR (2016). The temporal dynamics of spoken word recognition in adverse listening conditions. J. Psycholinguist. Res..

[47] Greenwood DD (1990). A cochlear frequency-position function for several species—29 years later. J. Acoust. Soc. Am..

[48] Best V, Carlile S, Jin C, van Schaik A (2005). The role of high frequencies in speech localization. J. Acoust. Soc. Am..

[49] Pulkki V (1997). Virtual sound source positioning using vector base amplitude panning. J. audio Eng. Soc..

