# Supplementary material for: Signal envelope and speech intelligibility differentially impact auditory motion perception
Source: Sci Rep. 2021 Jul 23;11:15117. doi: 10.1038/s41598-021-94662-y (PMC8302594; doi:10.1038/s41598-021-94662-y)
Supplement: Supplementary file 1 — Supplementary Information. [file 41598_2021_94662_MOESM1_ESM.pdf]

Signal envelope and speech intelligibility differentially impact auditory motion perception

Michaela Warnecke and Ruth Y. Litovsky

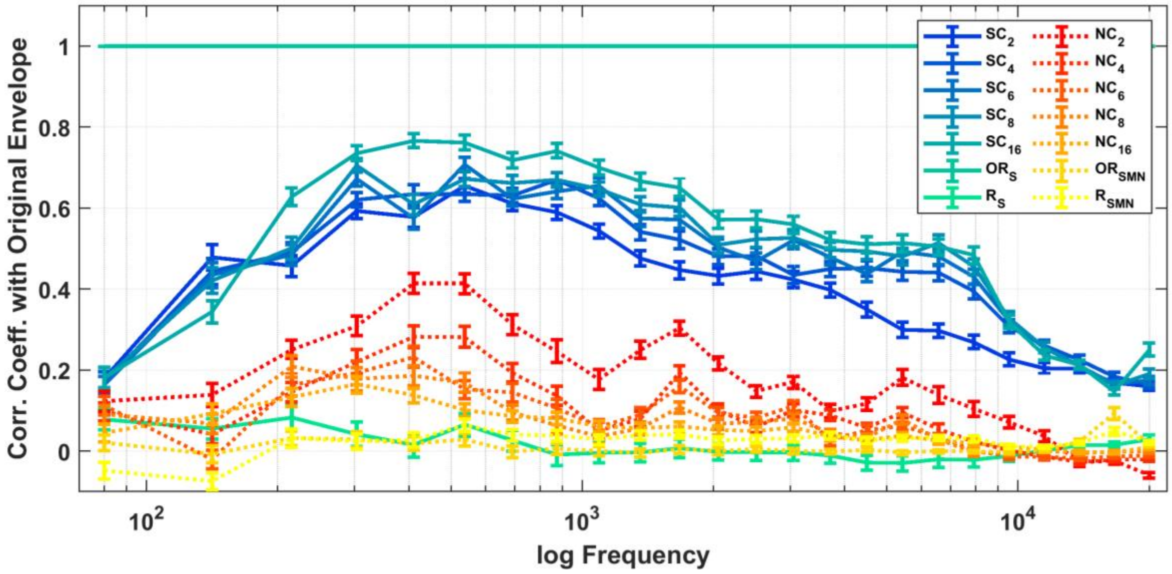

Supplementary Figure S1: Mean correlation coefficient (± 95% CI) between the original envelope (OR_S) at each output of a 24 gammatone auditory filter simulation and the reconstructed envelopes for each stimulus condition (legend). Means are calculated across simulation results for all 420 stimuli.

Stimuli for this experiment have been created using the available code library by Smith et al. (2002):

Smith, Z. M., Delgutte, B., & Oxenham, A. J. (2002). Chimaeric sounds reveal dichotomies in auditory perception. *Nature*, *416*(6876), 87-90.

The simulation has been prepared using the Two!Ears computational framework for modeling, available here: http://twoears.eu.

Winter, F., Wierstorf, H., Raake, A., & Spors, S. (2017, May). The two! ears database. In *Audio Engineering Society Convention 142*. Audio Engineering Society.

The analysis structure is modeled after Gilbert and Lorenzi's (2006) illustration of envelope recovery:

Gilbert, G., & Lorenzi, C. (2006). The ability of listeners to use recovered envelope cues from speech fine structure. *The Journal of the Acoustical Society of America*, *119*(4), 2438-2444.
